# Long‐Duration and Non‐Invasive Photoacoustic Imaging of Multiple Anatomical Structures in a Live Mouse Using a Single Contrast Agent

**DOI:** 10.1002/advs.202202907

**Published:** 2022-08-17

**Authors:** Anjul Khadria, Chad D. Paavola, Yang Zhang, Samuel P. X. Davis, Patrick F. Grealish, Konstantin Maslov, Junhui Shi, John M. Beals, Sunday S. Oladipupo, Lihong V. Wang

**Affiliations:** ^1^ Caltech Optical Imaging Laboratory Andrew and Peggy Cherng Department of Medical Engineering California Institute of Technology Pasadena CA 91125 USA; ^2^ Lilly Research Laboratories Eli Lilly and Company Lilly Corporate Center Indianapolis IN 46285 USA; ^3^ Lilly Research Laboratories Eli Lilly and Company Lilly Biotechnology Center San Diego CA 92121 USA; ^4^ Caltech Optical Imaging Laboratory Department of Electrical Engineering California Institute of Technology Pasadena CA 91125 USA

**Keywords:** contrast agents, lymphatic imaging, nerve imaging, photoacoustic imaging

## Abstract

Long‐duration in vivo simultaneous imaging of multiple anatomical structures is useful for understanding physiological aspects of diseases, informative for molecular optimization in preclinical models, and has potential applications in surgical settings to improve clinical outcomes. Previous studies involving simultaneous imaging of multiple anatomical structures, for example, blood and lymphatic vessels as well as peripheral nerves and sebaceous glands, have used genetically engineered mice, which require expensive and time‐consuming methods. Here, an IgG4 isotype control antibody is labeled with a near‐infrared dye and injected into a mouse ear to enable simultaneous visualization of blood and lymphatic vessels, peripheral nerves, and sebaceous glands for up to 3 h using photoacoustic microscopy. For multiple anatomical structure imaging, peripheral nerves and sebaceous glands are imaged inside the injected dye‐labeled antibody mass while the lymphatic vessels are visualized outside the mass. The efficacy of the contrast agent to label and localize deep medial lymphatic vessels and lymph nodes using photoacoustic computed tomography is demonstrated. The capability of a single injectable contrast agent to image multiple structures for several hours will potentially improve preclinical therapeutic optimization, shorten discovery timelines, and enable clinical treatments.

## Graphical abstract

This work shows simultaneous imaging of blood and lymphatic vessels, peripheral nerves, and sebaceous glands in a live mouse for several hours through photoacoustic microscopy using sulfo‐cy7.5 dye‐labeled IgG4 antibody as a single contrast agent. The authors also perform photoacoustic computed tomography to visualize deep (0.4 cm) lymphatic vessels in a live mouse for up to 3 h.

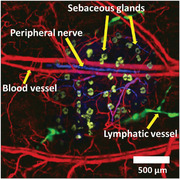

## 1 Introduction

Concurrent long‐duration live animal imaging of different anatomical structures, including blood and lymphatic vessels, peripheral nerves, and sebaceous glands can underpin an improved understanding of disease progression, study effects of therapeutics, and guide molecular optimization in preclinical models.^[^ ^1^, ^2^, ^3^ ^]^ Fluorescence microscopy has been shown to simultaneously image the abovementioned anatomical structures using mice genetically engineered with fluorescent proteins. However, such methods are expensive, time‐consuming, and are not currently translatable clinically.^[^ ^4^, ^5^, ^6^ ^]^ The discovery of lymphatic vessels in the mouse and human brain has made the need for long‐duration simultaneous imaging of lymphatic vessels and other anatomical structures at cellular‐level resolution ever more critical to better understand brain‐related diseases and optimize new therapies.^[^ ^7^, ^8^ ^]^ Previous work involving preclinical imaging of lymphatic vessels has used standalone dyes such as Evans blue or indocyanine green (ICG), which are not photostable, get absorbed by the blood vessels, and cannot be used for long‐duration imaging.^[^ ^3^, ^9^, ^10^ ^]^ The clearance of these dyes in less than 30–60 min after injection is well documented in both preclinical and clinical studies.^[^ ^10^, ^11^ ^]^ Deep lymphatic vessels in mice have been previously imaged using near‐infrared fluorescence imaging; however, those studies did not image the blood vessels simultaneously and utilized Evans blue or ICG.^[^ ^12^, ^13^ ^]^ Previous photoacoustic imaging‐based studies involving simultaneous imaging of blood and lymphatic vessels have used Evans blue or ICG, or required up to five wavelengths, which is expensive and complex.^[^ ^3^, ^10^, ^14^, ^15^ ^]^ Peripheral nerves are an integral part of the nervous system linking the brain to the rest of the body. Imaging of peripheral nerves is of utmost importance, for example, to study the nervous system to develop strategies to prevent accidental injuries during surgeries.^[^ ^16^, ^17^ ^]^ Previous work on photoacoustic imaging of peripheral nerves has been limited to only ex vivo studies.^[^ ^18^, ^19^ ^]^ Studies reporting in vitro and ex vivo multi‐feature photoacoustic imaging have pushed the limits of bioimaging; however, no in vivo studies have been reported that demonstrate simultaneous photoacoustic imaging of lymphatic vessels, peripheral nerves, and sebaceous glands along with blood vessels.^[^ ^20^, ^21^, ^22^ ^]^ Contrast agents that can facilitate long‐duration and non‐invasive in vivo photoacoustic imaging of multiple anatomical structures can benefit several preclinical and potentially clinical physiological studies and diagnoses such as peripheral neuropathy, lymphoma, vasculitis, and sebaceoma.^[^ ^23^, ^24^, ^25^ ^]^

Several photoacoustic‐based contrast agents have been reported in recent years; however, most agents are based on tumor imaging using organic or inorganic small molecules or nanoparticles.^[^ ^26^, ^27^, ^28^ ^]^ Notably, little or no advancement has taken place in contrast agents for long‐duration photoacoustic imaging of lymphatic vessels and other anatomical structures such as peripheral nerves and sebaceous glands.

**Figure 1:**
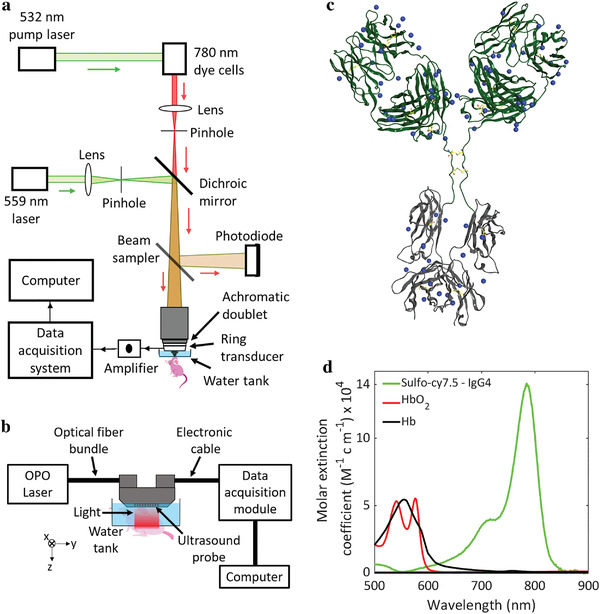
a) Schematic of the OR‐PAM system. b) Schematic of the linear array‐based PACT system with a view from the elevational direction (*x*‐axis) of the ultrasound probe emphasizing the imaging of the mouse hind limb. c) Scheme of IgG4 isotype control antibody with the available sites (as blue dots) for the conjugation of sulfo‐cy7.5 dye. d) Light absorption spectra of sulfo‐cy7.5 dye‐labeled IgG4 antibody, oxygenated hemoglobin (HbO_2_), and deoxygenated hemoglobin (Hb).

In this report, we use optical‐resolution photoacoustic microscopy (OR‐PAM) (**Figure** **1**a)^[^ ^29^ ^]^ to simultaneously image blood and lymphatic vessels along with peripheral nerves and sebaceous glands at cellular‐level resolution in the mouse ear skin. We used photoacoustic imaging to visualize blood (label‐free); however, to image lymphatic vessels, sebaceous glands, and axonal peripheral nerves, we subcutaneously injected the near‐infrared light absorbing sulfo‐cy7.5 dye‐labeled monoclonal human IgG4 isotype control antibody. In addition, we injected the dye‐labeled antibody in the mouse hind‐paw to observe its deep medial lymphatic vessel and lymph node through a hand‐held photoacoustic computed tomography (PACT) probe (Figure 1b).^[^ ^30^ ^]^ Dye‐labeled antibodies are used widely to fluorescently label and image anatomical structures through epitope bindings; however, in this report, we use an IgG4 isotype control antibody, which does not have any specific binding epitope.^[^ ^31^, ^32^ ^]^

## 2 Results and Discussion

### 2.1 Dye‐Labeling of the IgG4 Isotype Control Antibody

The IgG4 antibody was labeled with the sulfo‐cy7.5 dye (Figure 1c) and characterized by SEC‐HPLC and MALDI mass spectrometry (see Experimental Section). The labeled antibody was found to have ≈4.5 dyes per molecule and exhibited size exclusion behavior consistent with a monomeric, well‐behaved antibody (Figure S1, Supporting Information). The antibody has no specific antigen binding and incorporates S228P/L234A/L235A sequence changes in the Fc region to reduce immune effector function.^[^ ^33^ ^]^ While the current study does not employ any specific paratope, antibodies with specific binding could alternatively be employed to image other anatomical structures offering tissue specificity advantage as the case may be. We further characterized the dye‐labeled IgG4 antibody with UV–vis spectroscopy and found that it has an absorption maximum at 780 nm with a molar extinction coefficient of around 140000 M^−1^ cm^−1^, which is orders of magnitude higher than that of both oxygenated and deoxygenated blood at the same wavelength (Figure 1d). A higher extinction coefficient at 780 nm will ensure high contrast signals from the dye‐labeled IgG4 antibody.

### 2.2 Long‐Duration OR‐PAM of Lymphatic Vessels in Mouse Ear

**Figure 2:**
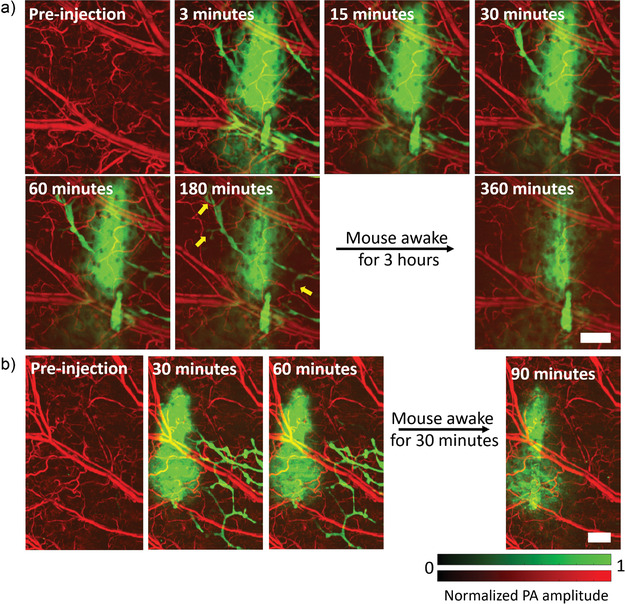
OR‐PAM of lymphatic vessels in mouse ear. a) Visualization of lymphatic vessels for up to 180 min upon injection of sulfo‐cy7.5 dye labeled IgG4 antibody. b) Imaging of the dye‐labeled IgG4 antibody performed in the mouse ear while keeping the mouse under anesthesia for a shorter period. Yellow arrows show lymphatic vessels lighting up. PA: photoacoustic. Scale bars: 500 µm.

We performed the OR‐PAM at 559 and 780 nm to visualize the blood and the dye‐labeled antibody, respectively, based on their absorption spectra (Figure 1d). We injected the dye‐labeled antibody (0.2 µL, 20 mg mL^−1^) into the mouse ear under anesthesia and performed photoacoustic imaging using OR‐PAM. We rely on the high molecular weight (≈147 kDa) of the antibody to drive lymphatic absorption at the point of injection since molecules greater than 20–25 kDa weight are predominantly absorbed by lymphatic vessels.^[^ ^34^, ^35^ ^]^ Antibodies, due to their large size, have a slow absorption rate from the subcutaneous injection site, which we utilize to perform long‐duration imaging.^[^ ^36^, ^37^ ^]^ The lymphatic vessels continuously absorbed the dye‐labeled antibody over the first 3 h of imaging, leading to the visualization of new lymphatic vessels while the mouse was under anesthesia (**Figure** **2**a, and Movies S1 and S2, Supporting Information). After the first 3 h of imaging, the mouse was allowed to recover from anesthesia and kept in an enclosure until the injection site was reimaged 6 h post injection. At 6 h, we did not observe any lymphatic vessels stained with the dye‐labeled antibody due to enhanced lymphatic clearance from the increased muscle‐mediated movement of the ear in the conscious state.^[^ ^38^ ^]^ To verify that enhanced absorption of the antibody occurs while the mouse was moving, we reduced the initial anesthesia time to 60 min and revived the mouse for only 30 min, then imaged it again under anesthesia (Figure 2b). Under these experimental conditions, we did not observe any lymphatic vessels around the injection site at 90 min.

### 2.3 Multiple Anatomical Structures Visualizations in Mouse Ear

**Figure 3:**
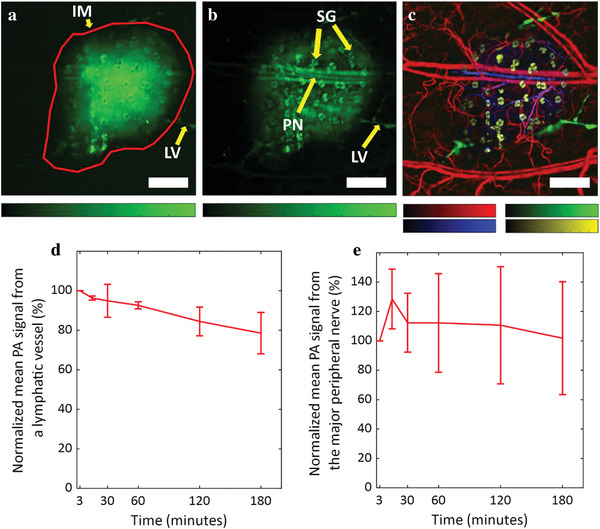
OR‐PAM of multiple anatomical structures. a) MAP image of the complete dye‐labeled antibody injected in mouse ear depicting the injected dye‐labeled antibody mass and the lymphatic vessels outside the mass. b) MAP image of the dye‐labeled antibody in mouse ear after inspection of 2D sections in the 3D volume to remove background signals. The sulfo‐cy7.5 dye‐labeled IgG4 antibody stains axonal peripheral nerves, sebaceous glands, and the major blood vessels inside the injected dye‐labeled antibody mass, and the lymphatic vessels outside the mass. c) Different anatomical structures labeled in pseudo colors through image segmentation. Blood vessels are colored as red (559 nm), lymphatic vessels as green (780 nm), sebaceous glands as yellow (780 nm), and axonal peripheral nerves as blue (780 nm). d) The mean photoacoustic signal from a single lymphatic vessel. e) The mean photoacoustic signal from the major nerve. Data represent mean ± standard deviation (*n* = 3). IM: injected mass; LV: lymphatic vessel; PN: peripheral nerve; SG: sebaceous gland. All color bars represent normalized photoacoustic amplitude and range from 0 to 1. Scale bars: 500 µm.

Our approach to visualizing multiple anatomical features is based on imaging the peripheral nerves and sebaceous glands inside the injected dye‐labeled antibody mass while visualizing the lymphatic vessels outside the mass. In our system, the 780 nm light that we used to detect the sulfo‐cy7.5 dye‐labeled IgG4 antibody has a depth of focus of about 300 µm, which covers the thickness of an average mouse ear (200–250 µm). The maximum amplitude projection (MAP) of the complete 3D photoacoustic image shows only the injected mass of the dye‐labeled antibody and the lymphatic vessels outside the mass (**Figure** **3**a). The sebaceous glands and peripheral nerves are not clearly visible due to excess background signals (from the dye‐labeled antibody) in the MAP image. We inspected 2D sections at various depths to avoid the excess dye‐labeled antibody background signals and distinctly visualize the axonal peripheral nerves and sebaceous glands (Figure 3b). We further performed image segmentation (see Experimental Section) to digitally label the different structures (Figure 3c). Peripheral nerves and arteries are aligned together in mouse skin, a feature that enabled us to identify the nerves in our images.^[^ ^6^, ^39^ ^]^ The circular structured sebaceous glands were noticeably visible in the images. The visualization of sebaceous glands in the mouse ear could be helpful in several types of skin studies.^[^ ^40^ ^]^ Many biological phenomena or disorders related to sebaceous glands, such as sebaceous adenoma, sebaceoma, sebaceous gland hyperplasia, sebaceous carcinoma, and folliculosebaceous cystic hamartoma, can be studied by imaging the size and shape of sebaceous glands as well as their effects on surrounding vessels.^[^ ^41^ ^]^ The major blood vessels in the mouse ear are also seen in Figure 3b, suggesting that the dye‐labeled IgG4 antibody encapsulated the vessels, creating a contrast as the antibody is too large to be absorbed through the pores of the blood microvasculature.

Upon quantitative comparison of the mean photoacoustic signal from a single lymphatic vessel that remains visible throughout the imaging time, we found that the photoacoustic signal does not fade away significantly (≈20% decrease) even 3 h after the injection under anesthetized conditions (Figure 3d). As mentioned above, the widely used lymphatic contrast agents such as ICG or Evans blue get cleared away in less than 1 h.^[^ ^10^, ^11^ ^]^ These results validate the efficacy of the large‐sized dye‐labeled monoclonal antibody for long‐duration lymphatic imaging. The peripheral nerves were also visible for up to 3 h (Figure 3e and Figure S2, Supporting Information) without a significant decrease in the mean photoacoustic signal. Note, the signal was detected after 50 days of initial injection (Figure S3, Supporting Information) without observing any local or systemic adverse effects in the mouse. This is notable because it infers that our dye‐antibody conjugate is not likely to cause any serious, acute toxicity. Monoclonal antibodies are widely used as therapeutic drugs and over 100 monoclonal antibodies have been approved by the FDA for clinical use.^[^ ^42^, ^43^ ^]^ The cyanine‐based dyes such as cy7 and cy7.5 and their conjugates with monoclonal antibodies have been used in multiple preclinical studies without observation of any adverse effects in the animals.^[^ ^44^, ^45^ ^]^

### 2.4 Long‐Duration Deep Lymphatic Vessel Visualization

**Figure 4:**
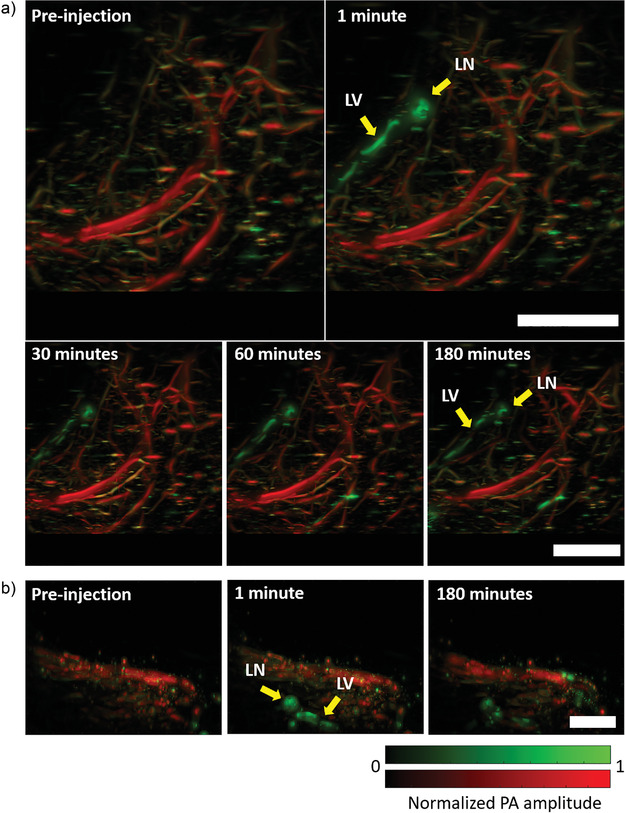
Long‐duration photoacoustic imaging of deep lymphatic vessels in mouse. a) Top‐view images of deep medial lymphatic vessel and lymph node (green), and blood (red) in mouse leg and thigh taken through PACT. b) Side‐view images of deep medial lymphatic vessel (green) and blood (red) in mouse leg and thigh taken through PACT. LV: lymphatic vessel; LN: lymph node; PA: photoacoustic. Scale bars: 0.4 cm.

We injected the dye‐labeled antibody in the mouse hind paw and performed imaging away from the site of injection through PACT at 780 and 920 nm in the leg and thigh areas where the deep medial lymphatic vessel and lymph node are located to observe them (**Figure** **4**a,b and Figure S4 and Movie S3, Supporting Information).^[^ ^12^ ^]^ Although the pre‐injection and post‐injection images at 780 nm were sufficient to establish the dye‐labeled antibody uptake by the lymphatic vessel and lymph node, we performed imaging at 920 nm to confirm that no blood leakage occurred during needle insertion while performing the injection. We chose 920 nm to perform imaging of blood because the extinction coefficient of hemoglobin in the blood in the NIR region (without overlapping with the absorption spectrum of the dye‐labeled IgG4 antibody) is highest at around 920 nm, which ensures more photoacoustic signal from the blood.^[^ ^46^ ^]^ The images in Figure 4 were processed by vessel segmentation (see Experimental Section). We performed imaging for up to 3 h following injection to continuously observe bright signals from the lymphatic vessels at a depth of 2–4 mm from the surface of the mouse skin, thus proving the long‐duration efficacy of the method (Figure 4b and Figure S4, Supporting Information). The dye‐labeled antibody does not absorb any light at 920 nm, but the blood absorbs significant light. We did not perform any multi‐feature imaging using PACT in deep tissues because of two reasons: 1) The sebaceous glands are present only in the skin for which high resolution‐based OR‐PAM is sufficient. 2) We have no pre‐information about the nerves in deep tissues to correctly identify them such as we had for mouse skin where the peripheral nerves and arteries are aligned together.^[^ ^6^, ^39^ ^]^

These results show that the dye‐labeled IgG4 antibody can be used for long‐duration photoacoustic imaging of superficial and deep lymphatic vessels. Long‐duration imaging of deep lymph nodes and lymphatic vessels could be a powerful tool for visualizing deep tumors during cancer metastasis.

## 3 Conclusions

In this study, we used IgG4 monoclonal antibody labeled with near‐infrared sulfo‐cy7.5 dye to perform photoacoustic imaging of multiple anatomical structures in mouse ear skin and deep medial lymphatic vessels in the leg and thigh. The slow clearance rate of the large‐sized antibody allows long‐duration photoacoustic imaging of the anatomical structures, which is otherwise difficult to perform with the range of existing contrast agents. The long‐duration multi‐anatomical structure labeling feature of the dye‐labeled antibody can be used in several types of preclinical studies involving studies of diseases and drug therapeutics. It also has the potential to be clinically translatable. The antibodies can be further designed to bind to specific antigens to enable long‐duration imaging of deep and shallow individual anatomical structures in vivo at high resolution, which is otherwise difficult to image due to a lack of sufficient injectable contrast agents, especially for photoacoustic imaging.

## 4 Experimental Section

### Sulfo‐Cy7.5 Dye Labeling of Human IgG4 Isotype Control Antibody

An IgG4 isotype antibody was labeled with Sulfo‐Cyanine‐7.5 dye using NHS ester chemistry (66320, Lumiprobe). The antibody was prepared at ≈10 mg mL^−1^ in 90 mm carbonate, 9 mm phosphate buffer with 125 mm sodium chloride at pH 8.3. The dye was dissolved in a one‐tenth volume of carbonate buffer immediately before adding to the antibody at a 10 equivalent excess and incubated at 25 °C for 4 h. The labeled antibody was isolated from the excess dye on a size exclusion chromatography column (Superdex S200, Cytiva) with a mobile phase of 1× PBS, pH 7.2 at 1 mL min^−1^. The degree of labeling was determined using MALDI‐MS to be ≈4.5 dyes per antibody. The antibody was concentrated to ≈30 mg mL^−1^ using a 15 mL spin concentrator (Millipore) with a 100 kDa MWCO membrane. Samples of labeled and unlabeled antibody (5–10 µg) were run on an HPLC (Agilent 1260 Infinity II) using an Agilent AdvanceBio SEC 300Å 2.7 mm column (PL1580‐3301, Agilent) at 1 mL min^−1^ in a mobile phase of 1× PBS; pH 7.2 (20012‐027, GIBCO) with peaks detected by absorbance at 214 nm. The total run time was 7 min.

### Measurement of the Extinction Coefficient of Sulfo‐Cy7.5 Dye‐Labeled IgG4 Antibody

The absorbance of the sulfo‐cy7.5 dye‐labeled IgG4 antibody (1 µm antibody, 4.5 dyes labeled per antibody) was measured using a UV–vis spectrophotometer (Cary 50 Bio, Varian) at 20 °C. The extinction coefficient was calculated using the Beer–Lambert law, *A* = *εCL* where *A* was the absorbance, *ε* was the molar extinction coefficient, *C* was the dye concentration, and *L* was the light path length (1 cm). The molar extinction coefficient values for oxygenated and deoxygenated blood were taken from the compilation of Scott Prahl.^[^ ^46^ ^]^

### Photoacoustic Microscopy System Design

A spherically focused ring‐shaped transducer (central frequency = 42 MHz, f‐number = 1.67, Capistrano Labs) was employed in the photoacoustic microscopy (PAM), which was equipped with two lasers of 559 and 780 nm optical wavelengths. Light pulses of both wavelengths were used to irradiate the same point successively with microseconds delay. The light beam from a 559 nm Nd:YAG laser (BX2II, Edgewave) was focused on a 254 µm diameter orifice (3928T991, McMaster‐Carr) for spatial filtering. A dye laser (Credo, Sirah) pumped by a 532 nm Nd:YAG laser (IS80‐2‐L, Edgewave GmbH) was employed to generate the 780 nm light beam. Styryl 11 dye (07980, Exciton) in 200 proof ethanol (MFCD00003568, Koptec) was circulated in the dye laser at 18 °C. The 780 nm light beam was combined with the 559 nm light beam through a dichroic mirror (M254C45, Thorlabs). Before the combination, the 780 nm beam was focused on a second pinhole (3928T991, McMaster‐Carr) for spatial filtering. A separate pinhole for the 780 nm light beam was used to increase its depth of focus (≈300 µm) with a lower resolution and also to correct for the focal length difference caused by light dispersion in the immersion liquid. An achromatic doublet (AC080‐020‐A, Thorlabs) was used to focus the combined beam on the sample. Some amount of light was collected by a photodiode (PDA36A, Thorlabs) with the aid of a beam sampler to correct for laser fluctuations. The animal was raster scanned using a stepper motor‐based 2D scanner (PLS‐85, Physik Instrumente) controlled by a customized LabVIEW program with an FPGA (PCIe‐7841, National Instruments). The data was acquired using a digitizer (ATS 9350, AlazarTech) at 500 MS s^−1^.

### Nano‐Liter Injection for Photoacoustic Imaging

31‐gauge needles (7803‐03, Hamilton) fitted in microliter syringes were used to inject antibody solutions at volumes of 0.10 and 0.2 µL in the mouse ear. To perform the rapid and controlled injection, the syringes were fitted onto a syringe dispenser (PB600, Hamilton). A 5 µL (7634‐01, Hamilton) syringe was used to perform the injections.

### Photoacoustic Computed Tomography System Design

The PACT system consisted of a laser source for optical illumination, an ultrasound linear array for recording the photoacoustic signals, a motor to perform a linear scan, a data acquisition (DAQ) module to digitize the signals, and a processing system for reconstructing the image (Figure 1b). An optical parametric oscillator (OPO) laser (SplitLight EVO III–100, Innolas) was used to deliver light at wavelengths of 780 and 920 nm to the target through an optical fiber bundle. The linear probe with a bandwidth of 13–24 MHz (MS 250, VisualSonics) was mounted on the stepper motor (PLS‐85, Physik Instrumente) and connected to a Verasonics Vantage 256 system (Verasonics). It featured a 14‐bit analog‐to‐digital converter dynamic range and sampling frequencies up to 62 MHz. The PACT system operated at 100 Hz in trigger mode (triggered by a laser) to acquire 600 frames in 6 s. The universal back‐projection algorithm was used to reconstruct the images.^[^ ^47^ ^]^

### Animal Experiments

All imaging experiments were performed on mice using protocols approved by IACUC at the California Institute of Technology. Hsd:Athymic Nude‐Fox1^nu^ mice aged 4–12 weeks (Envigo) were used in all experiments while maintaining their body temperatures at 37 °C during imaging. All the mice were imaged under isoflurane anesthesia (1.25–1.50% isoflurane in the air at a flow rate of 1 L min^−1^).

*OR‐PAM*: The mouse ear was imaged at a 4 kHz A‐line rate with a fast axis (2.5 µm step size, 1100 steps) and a slow axis (5 µm step size, 800 steps), resulting in a total scanning time of 220 s. To image the dye‐labeled IgG4 antibody formulations in a mouse ear, a pre‐injection image of the mouse ear was taken before performing a sub‐microliter injection of the required antibody formulation. Then images were taken at 3, 15, 30, 60, and 180 min time points, during which the mouse was maintained under anesthesia. The imaging time‐point was defined as the mid‐point between imaging start and finish. After 180 min, the mouse was awakened and kept in its cage with food and water. The mouse was anesthetized and reimaged at 6 h time point post injection.

*PACT*: The 3D images were acquired by scanning the ultrasound probe (step size = 50 µm) for a total time of 6 s. The backside of the mouse leg and thigh regions where the deep medial lymphatic vessel was located was imaged using light with wavelengths of 920 and 780 nm. Then the dye‐labeled antibody (50 µL, 20 mg mL^−1^) was injected into the hind‐paw and the image was immediately acquired (within 1 min). Subsequent images were acquired at 15, 30 min, 1, 2, and 3 h after injection during which the mouse was maintained under continuous anesthesia at 37 °C.

### Image Segmentation

To segment the different anatomical structures, a series of operations were applied to all sections where relevant features were visible. Total variance denoising was first applied,^[^ ^48^ ^]^ and then Gaussian filters and morphological top‐hat filters were used to select structures of relevant scale. The sebaceous glands were masked in the first image using a Canny edge detection algorithm^[^ ^49^ ^]^ to detect the boundaries of the glands, which were filled with a morphological closing, and cleaned with a morphological dilation. The lymph vessels were isolated by similarly masking the central absorption region and using the complimentary mask. After the initial contrast‐enhancing operations, the peripheral nerves and the sebaceous glands in the second image were manually labeled.

### Quantification of Mean Photoacoustic Signal from the Lymphatic Vessel and the Peripheral Nerve

The areas covered by the major peripheral nerve or a lymphatic vessel (that was visible throughout the time series) in the MAP images were roughly selected (after passing the images through an average filter of size 3 × 3 pixels). Then the images were thresholded by the summation of the mean and three times the standard deviation of the background amplitude to segregate photoacoustic signals from the noise. The contrast to noise ratio of resultant photoacoustic amplitudes was calculated to acquire the mean photoacoustic signal from the peripheral nerve or the lymphatic vessel.

### Vessel Segmentation for PACT Images

Post‐processing of reconstructed PACT volumes was performed with MATLAB (Mathworks). Vessels were segmented using a Hessian‐based multiscale vessel enhancing operator.^[^ ^50^ ^]^ The eigenvalues of the Hessian matrix (|*λ*_1_| ≤ |*λ*_2_| ≤ |*λ*_3_|) gave a measure of the local curvature of the volume, which could be used to determine the probability that a pixel was part of a vessel‐like structure. The Hessian at a volume *P* at voxel *x* and scale *σ* is defined by Equation (1),

(M1) $$H\;\left(x,\sigma \right)=\sigma^{2}\;P\left(x\right)*\frac{\partial^{2}G_{\sigma }\left(x\right)}{\partial x^{2}}$$

where *G*_*σ*_ was a Gaussian kernel with a standard deviation, *σ*. The vessel segmentation operator was then given by Equation (2),

(M2) $$\begin{array}{ccc} & & V\;\left(x,\sigma \right)=\\ & & \left\{\begin{array}{c} 0\;\mathrm{if}\;\lambda_{2}>0\;\mathrm{or}\;\lambda_{3}>0\\ \left(1-\exp\left(-\frac{R_{a}^{2}}{2a^{2}}\right)\right)\left(\exp\left(-\frac{R_{b}^{2}}{2b^{2}}\right)\right)\left(1-\exp\left(-\frac{S^{2}}{2c^{2}}\right)\right)\;\mathrm{otherwise} \end{array}\right.\; \end{array}$$

where $S=\sqrt{\lambda_{1}^{2}+\lambda_{2}^{2}+\lambda_{3}^{2}}$ was a measure of the total curvature at *x*, $R_{a}=\frac{|\lambda_{2}|}{|\lambda_{3}|}\;$discriminated between vessel‐like and plate‐like structures, and $R_{b}=\frac{|\lambda_{1}|}{\sqrt{|\lambda_{2}\left|\right|\lambda_{3}|}}\;$discriminated sphere‐like structures. The parameters were set as *a* = 0.5, *b* = 0.5, *c* = max *λ*_3_/2. Scale invariant vessel segmentation was performed by taking the maximum of *V*(*x*, *σ*) for *σ* between 40 and 360 µm.

The processed 780 and 920 nm vessel maps were then registered. The 920 nm segmented vessels were used to mask the dye present in the vasculature. Maximum projections were displayed on a logarithmic scale.

### Statistical Analysis

For the quantification of photoacoustic amplitudes from the lymphatic vessel and peripheral nerve in Figure 3d,e, the data at different time points were first normalized with respect to the photoacoustic amplitudes at 3 min. To perform normalization, the mean of the signal at all the time points was divided by the mean at 3 min and then multiplied by 100 to calculate the percentage of the photoacoustic amplitude with respect to the initial time point (i.e., at 3 min). The data in Figure 3d,e represented mean ± standard deviation. Data from the images of three different mice were used for the analysis. All the analysis was performed on MATLAB (R2021b, Mathworks).

## Conflict of Interest

A.K., Y.Z., S.P.X.D., and J.S. declare no conflict of interest. C.D.P., P.G., J.M.B., and S.S.O. are employees and stockholders of Eli Lilly and Company. L.V.W. and K.M. have financial interests in Microphotoacoustics, Inc., CalPACT, LLC, and Union Photoacoustic Technologies, Ltd, which did not support this work.

## Author Contributions

A.K., C.D.P., J.M.B., S.S.O., and L.V.W. conceived the project and the ideas. C.D.P. and A.K. designed the chemistry and parameters for dye labeling. P.G. labeled the antibody with the dye and characterized them. P.G. and A.K. prepared the antibody and dye buffer solutions. A.K. and K.M. designed and built the scanning photoacoustic microscope. A.K. designed and performed all the experiments, and analyzed and interpreted the data. Y.Z. designed the PACT system and data reconstruction algorithm. A.K. and Y.Z. performed the PACT experiments. S.P.X.D. performed the image segmentation for the PAM data and vessel segmentation for the PACT data. J.S. wrote the LabVIEW software for photoacoustic data acquisition. L.V.W., S.S.O., and J.M.B. supervised the project. A.K. wrote the manuscript. C.D.P., Y.Z., S.P.X.D., J.M.B., S.S.O., and L.V.W. contributed to writing the manuscript.

## Supporting information

Supporting Information

Supplemental Movie 1

Supplemental Movie 2

Supplemental Movie 3

## Data Availability

The data that support the findings of this study are available from the corresponding author upon reasonable request.
