# Supplementary material for: Long‐Duration and Non‐Invasive Photoacoustic Imaging of Multiple Anatomical Structures in a Live Mouse Using a Single Contrast Agent
Source: Adv Sci (Weinh). 2022 Aug 17;9(28):2202907. doi: 10.1002/advs.202202907 (PMC9534965; doi:10.1002/advs.202202907)
Supplement: Supplementary file 1 — Supporting Information [file ADVS-9-2202907-s004.pdf]

ADVANCED SCIENCE
Open Access

Supporting Information

for *Adv. Sci.*, DOI 10.1002/advs.202202907

Long-Duration and Non-Invasive Photoacoustic Imaging of Multiple Anatomical Structures in a Live Mouse Using a Single Contrast Agent

Anjul Khadria, Chad D. Paavola, Yang Zhang, Samuel P. X. Davis, Patrick F. Grealish, Konstantin Maslov, Junhui Shi, John M. Beals, Sunday S. Oladipupo* and Lihong V. Wang**

Supplementary information

Long-duration and non-invasive photoacoustic imaging of multiple anatomical structures in a live mouse using a single contrast agent

Anjul Khadria[1], Chad D. Paavola[2], Yang Zhang[1], Samuel P. X. Davis[1], Patrick Grealish[2], Konstantin Maslov[1], Junhui Shi[1], John M. Beals[3]*, Sunday S. Oladipupo[2]*, Lihong V. Wang[1]*

[1]Caltech Optical Imaging Laboratory, Andrew and Peggy Cherng Department of Medical Engineering, Department of Electrical Engineering, California Institute of Technology, Pasadena, California, 91125, USA

[2]Lilly Research Laboratories, Eli Lilly and Company, Lilly Corporate Center, Indianapolis, Indiana, 46285, USA

[3]Lilly Research Laboratories, Eli Lilly and Company, Lilly Biotechnology Center, San Diego, California, 92121, USA

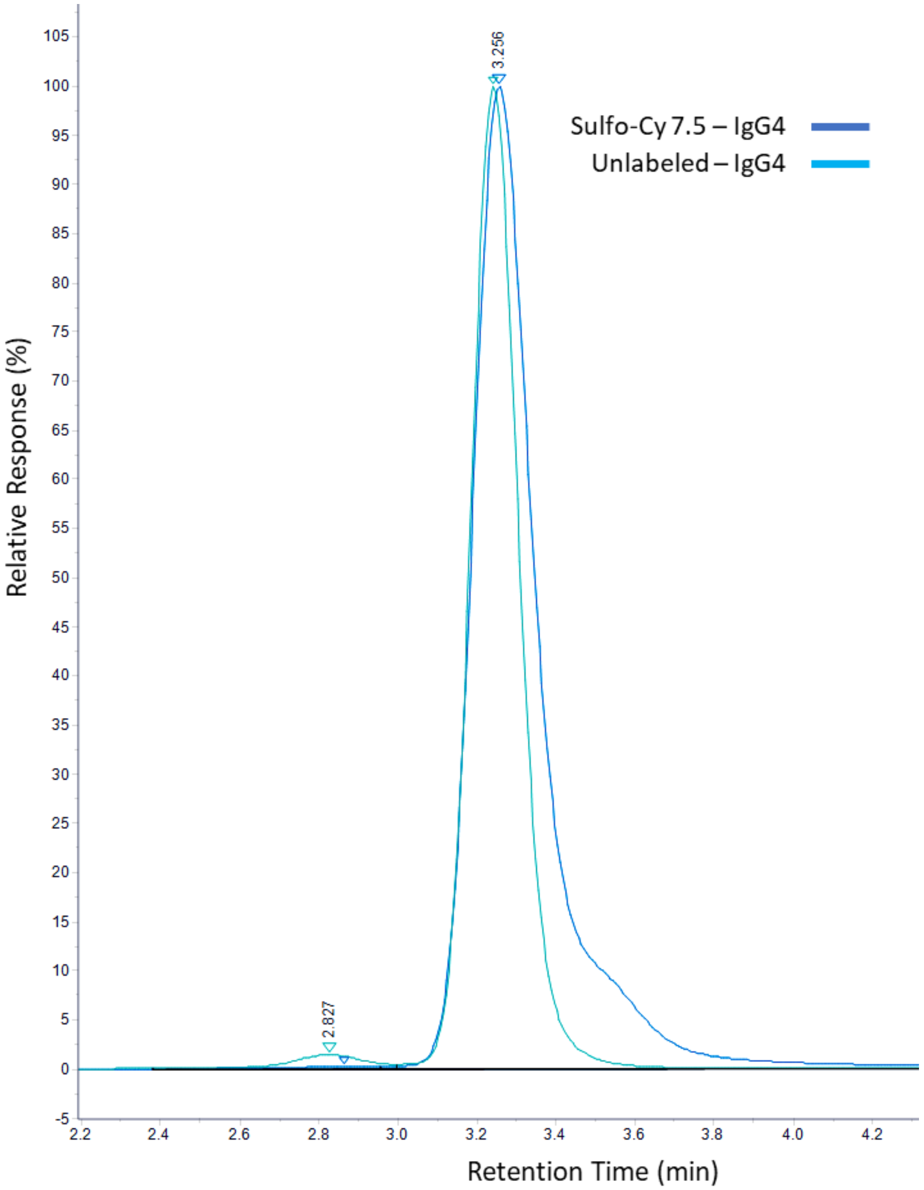

Figure S1: Overlay of sulfo-cy7.5 labeled (light blue) and unlabeled (dark blue) IgG4 isotype control antibody.

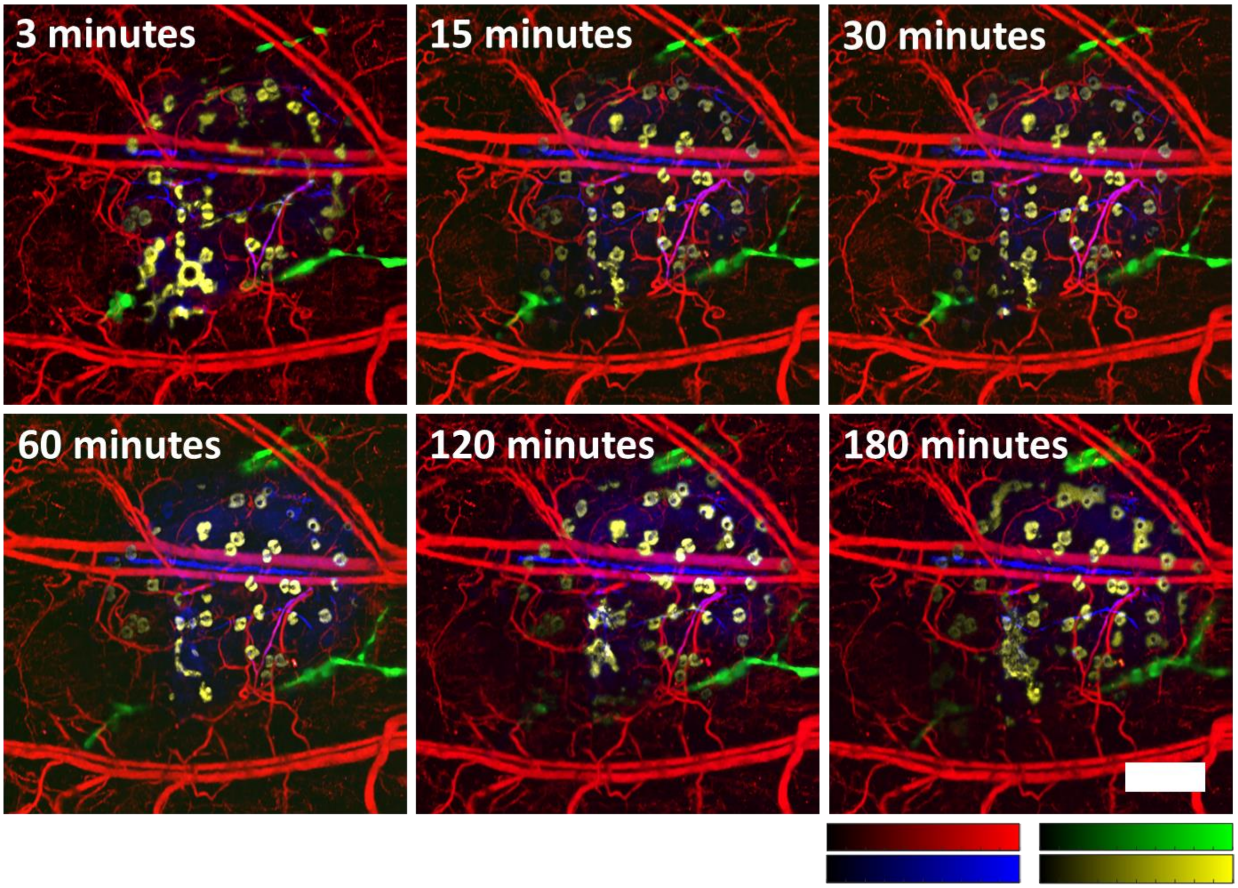

Figure S2: Long-duration imaging of multiple biological features in mouse ear skin after injection of dye-labeled monoclonal IgG4 isotype control antibody. All color bars represent normalized photoacoustic amplitude and range from 0 to 1. Scale bar, 500 µm.

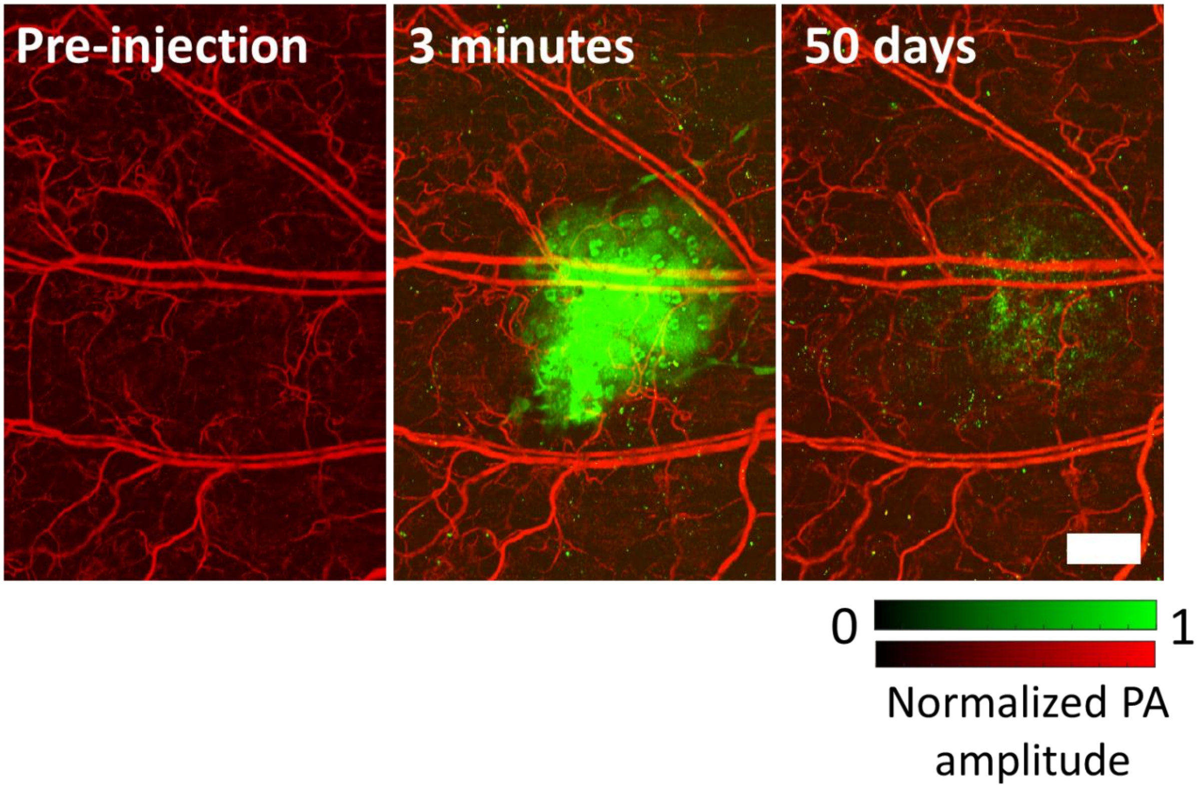

Figure S3: Detection of signals from dye-labeled IgG4 antibody even after 50 days of injection confirming its slow clearance rate. Scale bar, 500 μm.

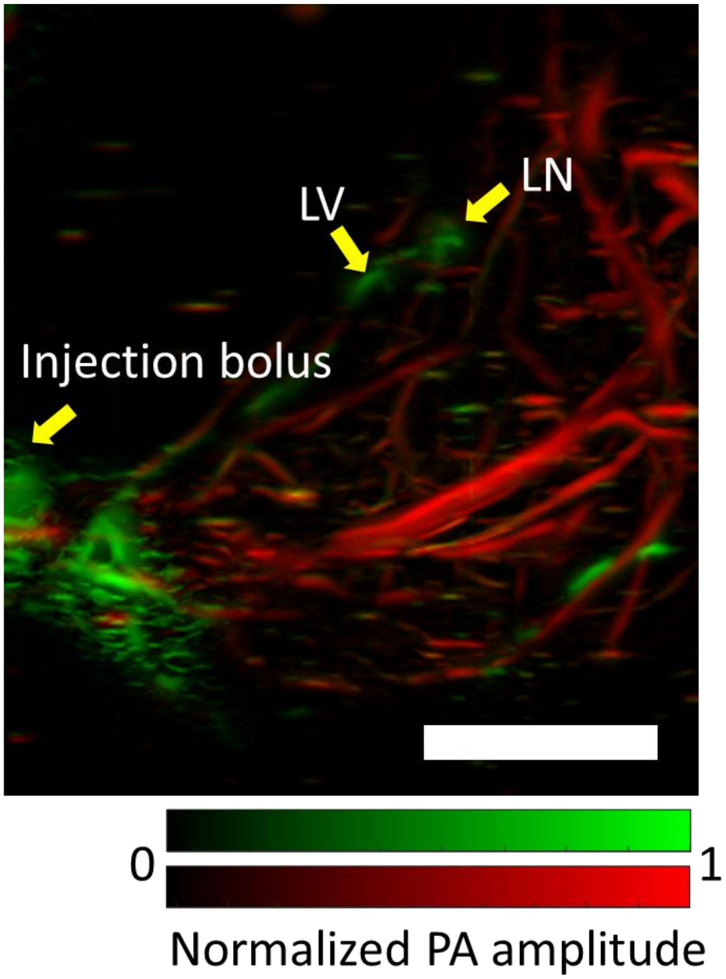

Figure S4: Visualization of deep medial lymphatic vessel and lymph node in the mouse leg and thigh 185 minutes after the injection of dye-labeled IgG4 antibody in mouse hind-paw using PACT. LV, lymphatic vessel; LN, lymphatic node; PA, photoacoustic. Scale bar, 0.4 cm.
